# Trends in mental health problems among Swedish adolescents: Do school-related factors play a role?

**DOI:** 10.1371/journal.pone.0300294

**Published:** 2024-03-08

**Authors:** Benti Geleta Buli, Peter Larm, Kent W. Nilsson, Charlotta Hellström-Olsson, Fabrizia Giannotta

**Affiliations:** 1 Department of Public Health Sciences, Mälardalen University, Västerås, Sweden; 2 Department of Public Health Sciences, Stockholm University, Stockholm, Sweden; 3 Center for Clinical Research, Uppsala University, Västmanland County Hospital, Västerås, Sweden; 4 Department of Neuroscience, Uppsala University, Uppsala, Sweden; University of Rome La Sapienza: Universita degli Studi di Roma La Sapienza, ITALY

## Abstract

**Aim:**

The aim of this study is to investigate the extent to which school-related factors, such as school liking, participation in decision-making, school-related parental support, teachers’ support, and school physical environment, explain trends in mental health problems. The problems considered are psychosomatic symptoms (PSS), depressive symptoms (DS), suicidal ideations (SI), and suicide attempts (SA) among Swedish adolescents of varying socioeconomic status (SES) from 2004 to 2020.

**Methods:**

We analyzed data collected through repeated cross-sectional surveys from 19,873 15-year-old students at schools in a county in Sweden. Boys and girls each constituted 50% of the participants. We fitted linear and logistic regression models to investigate associations between the school-related factors and trends in mental health problems.

**Results:**

Increased school-related parental support and school liking were cross-sectionally associated with decreased PSS, DS and SI, with school liking also associated with decreased SA. Conducive school physical environment was also found to be cross-sectionally associated with lower PSS and DS scores. Over time, mental health problems have shown a general increase among adolescents in the low SES group and a decrease among those in the high SES group. While school-related factors explained the improvement in mental health in the high SES group, we found such association only between parental support trends in PSS and DS, along with participation and trends in SA over time among adolescents in the low SES group.

**Conclusions:**

The results show that school-related factors play significant roles in influencing adolescent mental health. The influence, however, varied across SES gradients over time. This suggests that working against inequities in school-related factors would help address inequities in mental health.

## Introduction

Mental health problems among adolescents are among the growing challenges. Increasing trends in adolescent mental health problems have been reported in Sweden [1], in several countries in Europe and North America, and in Australia, New Zealand, Israel and China [2, 3] in recent decades. The reasons for these increasing trends are still largely unexplained.

Schools may offer an important context that influences adolescent mental health for various reasons. First, adolescents spend plenty of their awake hours at school [4], implying that a great deal of their life experiences occur at school. Second, school environments are ideal for social and cognitive development, which are key factors in mental health [5, 6]. Third, school-related social supportive arrangements are related to mental health either by directly providing emotional comforts or other necessary supports or by neutralizing the effects of school-related stress [7]. These traits position schools within the framework of living and working conditions in the social determinants of health model [8, 9], which impacts health, including mental well-being. Therefore, within this context, this study focuses on the influence of factors that pertain to school environment, such as student’s school liking, participation in decisions that affect school affairs, the support students receive from parents and teachers, and the conduciveness of the school’s physical environment, especially classrooms.

Several studies, for example, have associated higher school-related support from parents and teachers with decreased risk of mental health problems [10, 11], while low or lack of support increased the risk [12]. Participation in decision-making at school, termed as participation hereafter, and school liking are also among the factors that are related to adolescent mental health. For instance, participation was found to boost development of personal sense of control, which enhances academic endeavors and health [13]. Another study associated adolescents’ school liking with their overall well-being [14]. The interrelation between school liking and participation, where higher school liking implies higher participation, such as making school rules, was also associated with better self-reported health [15]. Other studies associated liking or disliking school with perception of amount of schoolwork [16] and interpersonal relationships in school [17]. Finally, physical characteristics of school facilities, such as cleanliness, space, and tranquility, are directly or indirectly associated with educational motivation [18], which in turn is associated with mental health [19]. To summarize, schools generally have various protective factors that are directly or indirectly associated with adolescent mental health. However, little is known about whether these factors are also related to trends in adolescent mental health problems.

The few studies that have considered trends in adolescent mental health problems [20–24] have focused on school stress or school demands as risk factors with little or no emphasis on the protective roles of school-related factors. Thus, it is not clear whether, or to what extent, changes in trends in mental health problems are associated with school-related factors that may play protective roles.

Another limitation of the literature lies in a lack of evidence on the effects of SES on the association between school-related factors and trends in adolescent mental health problems. In a recent study, we found different trends in mental health problems for different SES groups, where the trends increased among adolescents with low SES and decreased among others [25]. This calls for a better understanding of whether school-related factors have changed differently for different SES groups and influenced the trends in adolescent mental health problems accordingly.

This study aims to investigate associations between school-related factors and trends in adolescent mental health problems during the period from 2004 to 2020 by splitting its samples into low and high SES groups. The study uses four indicators of mental health: problems of varying degrees of severity, namely psychosomatic symptoms (PSS), depressive symptoms (DS), suicidal ideations (SI) and suicide attempts (SA), since mental problems may be influenced differently by different factors [26] and have varying trends [27]. The school-related factors considered include school liking, participation, school-related parental support, teachers’ support, and school physical environment. This study will first present trends in mental health problems and school-related factors from 2004 to 2020, separately for low and high SES groups. Second, it will explore cross-sectional associations between school factors and mental health problems over this period. Third, it will investigate whether school-related factors are associated with trends in mental health problems. To accomplish this, two approaches will be considered: 1) the exposure hypothesis, which suggests that trends in mental health problems could be explained by changes in mean scores of school-related factors over time (see [21] and [28]); 2) the vulnerability hypothesis, which suggests that trends in mental health problems could be explained by variations in the strength of associations between school-related factors and mental health outcomes [21]. Both hypotheses will be examined to discern the associations between school-related factors and trends in mental health problems for low and high SES groups.

## Methods

### Participants

We used data from the Survey of Adolescent Life in Västmanland (SALVe) project that has collected information since 1995 from adolescents in this Swedish county. The project’s surveys were conducted every two to three years in all secondary and upper secondary schools in the county to monitor the psychosocial health of the adolescents. Students in special schools and those with insufficient Swedish language skills were not included. The surveys were paper based until 2020 when online data collection was started. It was then completed in 1 hour during class hours under the supervision of teachers. Information on the data collection process in this project has been reported on in a previous study [25]. We analyzed data from a total of 19,873 students in Grade 9 (15-year-old) in all except special schools in the county between 2004 and 2020. The response rates ranged from 75% in 2017 to 87% in 2014 with an overall average rate of 80.4%. Boys and girls each constituted 50% of the participants, and 84.8% of the participants belonged to the high SES group. However, there was an irregularity in the data availability over the study period (2004–2020), causing each outcome to have a different time frame. Accordingly, we analyzed trends in PSS from 2004 to 2014, DS from 2004 to 2012, SI from 2004 to 2012, and SA from 2004 to 2020 (S1 Table in S1 File). This resulted in varying sample sizes between the four outcomes. The Suicide Attempt (SA) question had the highest missing proportion at 9% because it was not asked in 2014, while year of survey had no cases missing. Because the missing proportion of cases is generally low, we employed listwise deletion of missing values, while we dropped the 2014 datapoint from the analysis of SA. As a result, n = 15,750 for PSS, n = 13,539 for DS, n = 12,972 for SI, and n = 16,073 for SA were included in the analyses (S2 Table in S1 File). Västmanland’s population is considered representative of the country on the basis of its distributions of education, employment, and income levels, as well as urban and rural settings [29].

#### Ethical considerations

As the surveys were completely anonymous, there was no requirement for ethical approval under Swedish law (Ethical Review Act 2003: 460). Nevertheless, informed consent was obtained from the participating students. In addition, parents/guardians were provided with prior information about the study and given the option to inform the schools if they did not wish for their children to participate.

### Measurement

***Psychosomatic symptoms (PSS)***: We used a scale constructed from summation of eight items that assessed adolescents’ experience of PSS during three months before each survey date. The items were headache, stomachache, pain in the shoulders or neck, pain in the back or hips, pain in hands/knees/legs/feet, difficulty in sleeping, feeling nervous, and feeling irritated. Participants’ responses ranged from never (0) to always (4) when asked if they had experienced any of them. An index variable was created from summation of these scores resulting in a total score ranging 0–32 points (α = 0.81, ranges 0.80–0.82) where higher scores indicate worse psychosomatic health. Use of such a scale was reported in a previous study [30].

***Depressive symptoms (DS)*:** We measured DS on the scale developed from 15 questions based on the Adolescent version of the Depression Self-Rating Scale (DSRS-A) [31, 32]. The questions were designed to ascertain if the adolescents had experienced any of the symptoms during the two weeks before data collection. We transformed these 15 single items into nine groups of DS based on the criteria of major depression in the Fourth-edition of the Diagnostic and Statistical Manual of Mental Disorders (DSM-IV) [33]. The possible responses were “yes” (1) or “no” (0). Finally, an index variable was created from summation of the symptoms with a total score ranging 0–9 points (α = 0.85, ranges 0.83–0.86) where higher scores indicate worse mental health.

***Suicidal ideations (SI)*** and ***Suicide attempts (SA)*** are categorical variables that concern whether the adolescents had experienced recurring thoughts of taking, or tried to take, their lives during the two weeks before the survey. The response alternatives were “no” (0) or “yes” (1).

***School liking***: the students were asked how much they liked being in school, and the responses ranged from “least” (1) to “most” (5).

***Participation in decision-making at school*, *termed in this paper as participation for readability purpose*,** is a single item independent variable that requires answers to questions of whether students are involved in activities that influence decisions in school, and the responses ranged from “never” (1) to “always” (5).

***School-related parental support*** is a scale composed of three items: a) my parents are prepared to help in cases of any problem at school, b) my parents encourage me to do well at school, and c) my parents help me with my school assignments in cases of difficulties. The responses ranged from “strongly disagree” (1) to “strongly agree” (5). The total score ranged 3–15, with higher values indicating higher school-related parental support (α = 0.82, ranges 0.79–0.84). An exploratory factor analysis (EFA) conducted to assess the construct validity showed factor loadings ranging 0.77–0.84 (S3 Table in S1 File).

***Teachers’ support***: this scale is also composed of three items: a) the teachers are good at encouraging students to think independently, b) the teachers strive to ensure that no student is bullied, and c) the teachers give me useful viewpoints on my schoolwork. The responses ranged from “strongly disagree” (1) to “strongly agree” (5). The total score ranged 3–15, in which higher values indicated higher teachers’ support (α = 0.82, ranges 0.75–0.86). The EFA showed factor loadings of 0.70–0.86 (S3 Table in S1 File).

***Positive school physical environment***: this scale is composed of three items: a) the classrooms are quiet and give peace of mind, b) there is adequate space in the classrooms, and c) the classrooms are clean and comfortable. The responses ranged from “strongly disagree” (1) to “strongly agree” (5). The total score ranged 3–15, where higher values indicate a more conducive school physical environment (α = 0.78, ranges 0.75–0.80). The factor loadings, ranging from 0.67 to 0.83, derived from the EFA, indicate a robust construct for the scale (S3 Table in S1 File).

***SES***: We used a 7-rung scale [32] to assess the adolescents’ perceptions of their families’ SES. The adolescents indicated their own family’s SES status by answering the following question: “Imagine society as a ladder. Families with the least money are at the bottom of the ladder while those with the most money are at the top. If you think about your family’s wealth compared to that of society at large, where would you place your family on the ladder?” The points ranged from zero (least money) to six (most money). The scale was dichotomized using standard deviations, where all ≤ -1 SD were categorized as low SES (0) and all >-1 SD as high SES (1). ***Sex*** was another covariate question asked, to which the participants responded as either a boy (0) or a girl (1).

### Statistical analysis

Three levels of analysis were conducted. The first step involved identifying time trends in both mental health problems and school-related factors by comparing mean scores or average probabilities (for categorical outcomes) of independent and outcome variables across each survey year. To conduct this comparison, we employed the Reverse Helmert contrast method in the general linear model [34] which contrasts the mean score or proportion in a particular survey year with the average of the mean scores or proportions from the preceding survey years. For instance, comparisons were made between 2006 and 2004, 2008 and the average of 2004 and 2006, 2010 and the average of 2004, 2006, and 2008. This process continued until mean scores or probabilities at the final survey year were compared with the average of all the preceding survey years.

For the other two analyses, we estimated linear trends in this study after testing curvilinear estimation and found that the data best fit the linear model. In our pursuit of selecting the best model, we fitted multilevel null models for all the outcomes and schools (as the second-level variable) and identified intraclass correlations (ICC) of <0.05. This cut-off point, recommended by previous studies, signifies the threshold above which multilevel models (MLM) need to be considered [35]. Other sources suggest a higher cut-off at 0.1 [36, 37]. As a result, we proceeded with single-level models. Separate trends for low and high SES groups were estimated to assess the distinct roles of SES in moderating the association between school factors and mental health problems. Linear regression was employed for continuous variables (PSS and DS), while logistic regression was used for categorical outcomes (SI and SA). For each SES group, we fitted two models. The first model (Model 1) investigated the cross-sectional associations between the outcomes and school-related factors. Model 2 estimated the strength of association between school-related factors and the trends in mental health problems over time, through the test of interaction terms between survey year and each school-related factor. In Model 1, we further examined the mediation effects of each school-related factor on the relationship between the survey year and the outcome variables, using the approach outlined in Hayes [38]. R-squared change and χ^2^ change in the Omnibus test of model coefficients were used for linear and logistic regressions respectively to test significance of differences between Models 1 and 2 in predicting the outcomes following addition of the interaction terms. We controlled for “sex” in both the linear and logistic regression models. SPSS version 28 was used for the data analyses. For the mediation analysis, we used the 4^th^ version of Hayes [38] process in SPSS.

## Results

**Fig 1:**
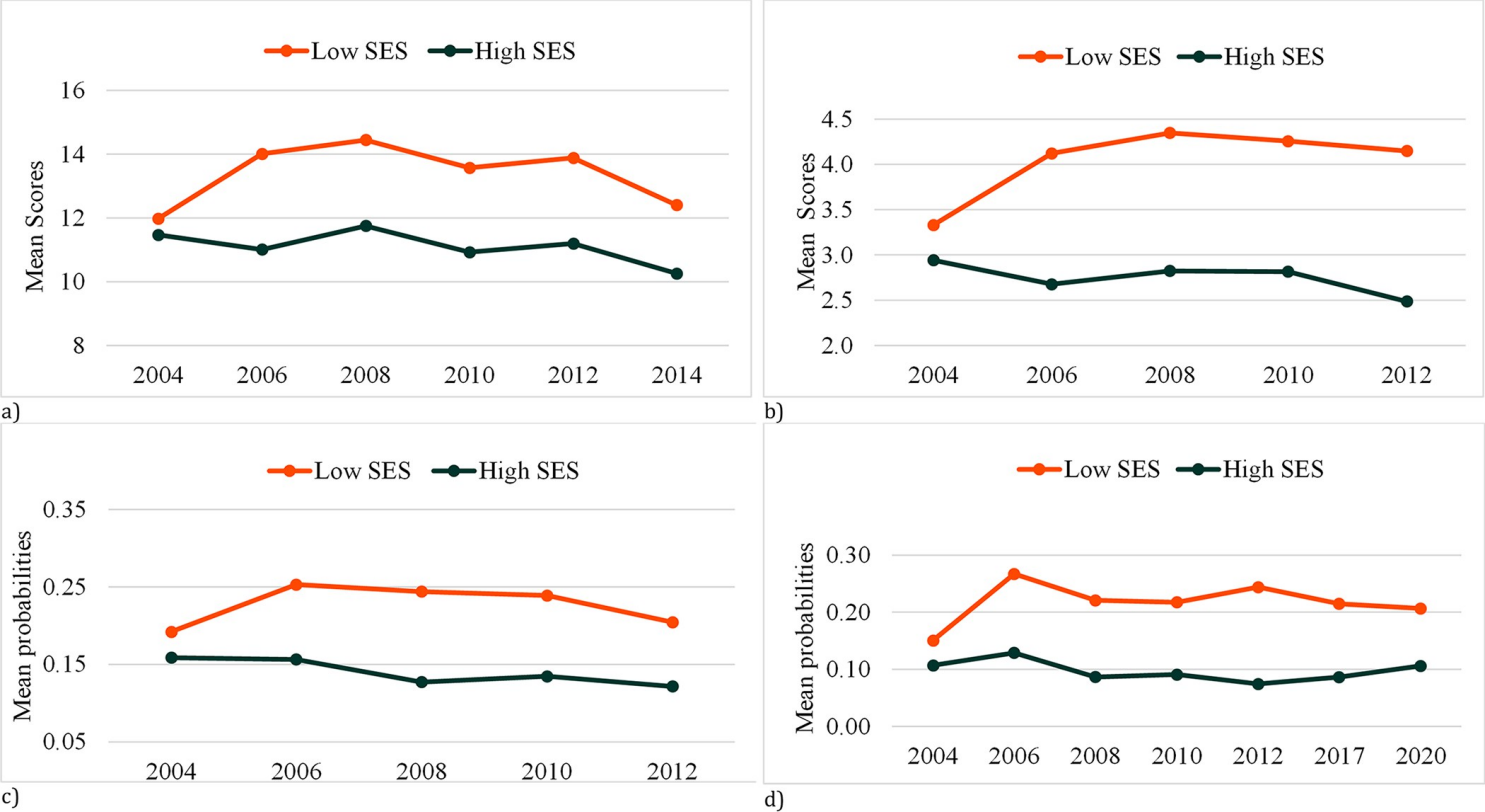
Distribution of mean scores of mental health problems by year of survey and SES. (a) psychosomatics symptoms, (b) depressive symptoms, (c) suicidal ideations, (d) suicide attempts.

Fig 1 shows distribution over time of mean scores of all indicators of mental health problems in this study. Additionally, it provides an overview of the distributions across participants’ subjective SES. As illustrated in Fig 1 and confirmed by the Reverse Helmert contrast, there is a generally increasing trend in mental health problems among adolescents in the low SES. The reverse Helmert contrasts show that mean scores of PSS and DS significantly increased between 2004 and 2008, then remained steady until 2014 when PSS began to decrease. SI and SA showed an increasing trend in this group between 2004 and 2006, after which they were stable. Conversely, there was a general decreasing trend, or stability in certain cases, among those in the high SES group, except for increases in PSS between 2006 and 2008, and in SA between 2004 and 2006. The details of the results are presented in the S2 Table of S1 File.

S1 Fig presents distributions of the mean scores of the school-related factors over time disaggregated by SES. Although there is an increase in the mean scores of the individual school-related factors over time, there are times when they have decreased generally, e.g., in 2006. Mean scores of school-related parental and teachers’ support again decreased in 2014. Mean scores of school liking, after a steady increase between 2006 and 2014, started falling thereafter. Participation increased overall except in 2014 and 2017 during which time it was not collected. The school physical environment showed a general improvement all the way through from 2004 to 2014. The mean scores of the school-related factors increased more in the high SES group (p < .001) than in the low SES group. However, there was no significant difference between the groups regarding changes in participation and school physical environment over time. The mean scores and SDs disaggregated by SES are presented in S4 Table of S2 File. Regarding SES, we found a significant increasing trend in SES mean scores from 3.53 in 2004 to 3.70 in 2020, with fluctuations over time. There was a decrease from 2004 to 2008, stability in 2010 and 2012, and a significant increase since 2014 (S5 Table in S2 File). The associations between these factors and adolescent mental health problems are presented below.

### Cross-sectional associations

The results from Model 1 in Table 1 indicate that school liking, parental support, teacher’s support, and the school physical environment exhibited significant negative associations with PSS in both the low and high SES groups. Similarly, all school-related factors, except teacher’s support and participation, demonstrated significant negative associations with DS in both SES groups (refer to Model 1 in Table 2). Table 3 (Model 1) shows that school liking and parental support showed significant negative associations with SI in both SES groups, while teacher’s support exhibited a negative association with SI solely in the low SES group. Finally, the findings in Table 4 (Model 1) reveal that only school liking showed a significant association with SA. Overall, the school-related factors in this study displayed negative associations with mental health problems at the cross-sectional levels.

**Table 1:** Linear regression results of the association between school-related factors and trends in PSS among adolescents aged about 15 years.

Variables	Low SES (a) (n = 2,446)		High SES (b) (n = 12,970)	
	Model 1(β)	Model 2 (β)	Model 1(β)	Model 2 (β)
School liking	-.172***	-.177***	-.156***	-.177***
Participation	.028	.030	.001	-.005
Parent support in school	-.152***	-.177***	-.083***	-.103***
Teachers’ support	-.053*	-.049	-.025*	-.023
School environment	-.110***	-.126***	-.089*	-.101***
Year of survey	.080***	.065**	.009	.018
Sex (female vs male)	.258***	.257***	.310***	.308***
School liking * year		-.009		-.056***
Participation* year		.008		-.019
Parent support * year		-.063*		-.043***
Teacher support * year		.007		-.003
School environment * year		-.037		-.037**

***p < .001

**P < .01

*p < .05

β = standardized coefficients; PSS = Psychosomatic symptoms; Scale: PSS was measured on a scale that ranged from 0 to 32.

**Table 2:** Linear regression results of the association between school-related factors and trends in DS among adolescents aged about 15 years.

Variables	Low SES (c) (n = 2,178)		High SES (d) (11,072)	
	Model 1(β)	Model 2 (β)	Model 1(β)	Model 2 (β)
School liking	-.182***	-.183***	-.188***	-.191***
Participation	.032	.031	-.016	-.015
Parent support in school	-.208***	-.214***	-.128***	-.140***
Teachers’ support	-.042	-.038	-.020	-.019
School environment	-.087***	-.093***	-.045***	-.042***
Year of survey	.103***	.090***	-.009	.010
Sex (female vs male)	.225***	.224***	.213***	.213***
School liking * year		-.005		-.039***
Participation* year		.004		-.006
Parent support * year		-.065**		-.076***
Teacher support * year		.025		-.017
School environment * year		-.033		-.002

***p<0.001

**p<0.01

*p<0.05

β = standardized coefficients; DS = Depressive symptoms; Scale: DS was measured on a scale that ranged from 0 to 9.

**Table 3:** Logistic regression results of the association between school-related factors and trends in SI among adolescents aged about 15 years.

Variables	Low SES (e) (2,178) OR (95% CI)		High SES (f) (11,072) OR (95% CI)	
	Model 1	Model 2	Model 1	Model 2
School liking	**.763** (.683, .853)	**.762** (.681, .853)	**.733** (.688, .781)	**.730** (.685, .778)
Participation	1.044 (.940, 1.160)	1.047 (.941, 1.165)	.964 (.913, 1.019)	.963 (.911, 1.018)
Parent support in school	**.890** (.859, .921)	**.890** (.859, .921)	**.920** (.902, .939)	**.913** (.895, .932)
Teachers’ support	.955 (.917, .995)	**.955** (.916, .996)	.992 (.970, 1.014)	.990 (.968, 1.012)
School environment	.967 (.924, 1.012)	.964 (.921, 1.010)	.988 (.965, 1.012)	.993 (.969, 1.017)
Year of survey	1.001 (.921; 1.087)	.991 (.902, 1.088)	**.938** (.898, .980)	**.933** (.892, .976)
Sex (female vs male)	**1.443 (**1.144, 1.820)	**1.443** (1.144, 1.820)	**1.308** (1.161, 1.473)	**1.311** (1.164, 1.477)
School liking * year		1.001 (.922, 1.087)		.984 (.938, 1.033)
Participation* year		1.013 (.937, 1.094)		.988 (.949, 1.029)
Parent support * year		.999 (.974, 1.026)		**.964** (.949, .979)
Teacher support * year		.994 (.964, 1.025)		.991 (.974, 1.008)
School environment * year		.987 (.953, 1.022)		1.013 (.995, 1.031)

OR = Odds ratio (*significant values at α <* .*05 are presented in bold*), CI = 95% Confidence Interval; SI = Suicidal ideations

**Table 4:** Logistic regression results of the association between school-related factors and trends in SA among adolescents aged about 15 years.

Variables	Low SES (g) (n = 2,560) OR (95% CI)		High SES (h) (n = 14,328) OR (95% CI)	
	Model 1	Model 2	Model 1	Model 2
School liking	**.662** (.599, .731)	**.680** (.605, .764)	**.632** (.595, .672)	**.603** (.564, .644)
Participation	.976 (.882, 1.080)	.893 (.794, 1.005)	.970 (.915, 1.030)	.978 (.918, 1.043)
Year of survey	1.022 (.967, 1.080)	1.035 (.969, 1.106)	**.960** (.931, .990)	**.932** (.900, .964)
Sex (female vs male)	**1.541** (1.230, 1.931)	**1.536** (1.225, 1.925)	**1.623** (1.423, 1.852)	**1.615** (1.416, 1.843)
School liking * year		1.025 (.974, 1.079)		**.945** (.919, .972)
Participation * year		**.925** (.877, .974)		1.004 (.977, 1.032)

OR = Odds ratio (*significant values at α <* .*05 are presented in bold*), CI = 95% Confidence Interval; SA = Suicide attempts

### Exposure hypothesis

The exposure hypothesis test indicated that school-related factors in this study did not exhibit any mediating effects on the relationship between the survey year and any of the mental health problems (PSS, DS, SI, or SA) among adolescents in the low SES group. However, in the high SES group, all the factors except participation were found to have mediated the association between the survey year and at least one mental health problem, demonstrating indirect negative effects on the scores of these problems. Hence, it can be inferred that the changes in mental health problems among adolescents in the low SES group might not be directly associated with changes in school-related factors over time. In the high SES group, on the other hand, improvements in school-related factors seem to be linked with a reduction in mental health problems over time (S6 Table in S2 File).

### Vulnerability hypothesis

#### Psychosomatic symptoms

The results from the interaction analyses show that associations between school-related factors and trends in PSS vary across gradients of SES over time. Particularly, in Model 2 in Table 1(a), it is evident that only parental support (β = -.063; p<0.05) has shown stronger protective association with trends in PSS over time in the low SES group. In the high SES group, the protective associations between school-related factors including parental support (β = -.043; p<0.001), school liking (β = -.056; p<0.001) and positive school physical environment (β = -.037; p<0.01) and trends in PSS grew stronger over time, as indicated in Model 2 in Table 1(b).

#### Depressive symptoms

The results indicate that the protective association between school-related parental support and trends in DS became stronger over time among adolescents in both low (β = -.065; p<0.01) and high (β = -.076; p<0.001) categories of SES. However, the protective association between school liking (β = -.039; p<0.001) and trends in DS grew stronger in the high SES group (Model 2 in Table 2).

#### Suicidal ideations

The results in Model 2 in Table 3(E) show that the strength of association between school-related factors and trends in SI did not change over time among adolescents in the low SES group. However, the protective association between school-related parental support and trends in SI grew stronger over time among adolescents in the high SES group (OR = .964; CI [.949, .979]) (Model 2 in Table 3(F)).

#### Suicide attempts

School liking and participation were the only school-related factors included in this model since data on the other school-related factors were not collected after 2014. The results in Model 2 in Table 4(g) indicate a stronger protective association between participation and trends in SA over time among adolescents in the low SES group (OR = .925; CI [.877, .974]). On the other hand, the protective association between school liking and trends in SA became stronger over time among those in the high SES group (OR = .945; CI [.919, .972]), as presented in Model 2 in Table 4(h).

We also conducted separate analysis for the period from 2004 to 2012, for which complete data were available for both the outcome and the independent variables. The findings in S7 Table of S2 File suggest that there was no difference between the results in this table and the original analysis in the association between school-related factors and trend in SA over time.

## Discussion

This study aimed at exploring whether school-related factors, i.e., school-related parental support, teachers’ support, school liking, participation, and school physical environment, are associated with trends in the mental health problems of adolescents of low or high SES groups. First, we found a general improvement in these factors over the years in both SES groups, while mental health problems became worse only for adolescents in the low SES group. It was found that parental support and school liking were cross-sectionally associated with decreasing mental health problems in both SES groups over the years. Conducive school physical environment was also cross-sectionally associated with decreased PSS and DS in both groups. To examine the associations between school-related factors and mental health problems among adolescents, both the exposure and vulnerability hypotheses were explored [21, 28]. The mediation analysis, aimed at testing the exposure hypothesis, indicated a notable role of school-related factors, except participation, in reducing mental health problems over time among adolescents in the high SES group but not among those in the low SES group. Nevertheless, in testing the vulnerability hypothesis, we observed varying strengths of associations between school-related factors and mental health problems over the years in both the low and high SES groups. Specifically, protective associations between only parental support and trends in PSS and DS grew stronger over time in both low and high SES groups. While the protective association between participation and trends in SA was stronger over time in the low SES group, the protective associations grew stronger over time between school liking and trends in PSS, DS, and SA only in the high SES group. These results suggest that SES may be the discriminating factor between the two groups, although the underlying mechanism remains unclear.

Among the major findings in this study is the cross-sectional association between school-related parental support and decreased trends in adolescent mental health problems, such as PSS and DS, regardless of SES differences, and SI among adolescents in the high SES group. This falls in line with previous studies that have related parental support to adolescents’ well-being and mental resilience either through providing emotional comforts, companionship, and tangible material supports or by giving relevant information that helps in the appraisal of situations to deal with stressful circumstances [5, 7, 39, 40]. The increased trend in parental and teacher supports in Sweden may be related, among other things, to two major policy circumstances. The first is the Swedish school system reform, one of whose aims was to increase the influences of parents, teachers, and students over the schools themselves [41]. Although the policy was introduced in 1992, it took full effect and expanded starting early 2000s [42] and this period of expansion of policy implementation aligns with our study period. The second is the subsequent policy directive that prompts schools to bolster partnerships between parents and teachers to help children attain desired academic achievement, well-being, and development [43]. All these items can play a role in boosting adolescents’ academic achievements, sense of support and confidence, and eventually contribute to buffering the stress from school demands [7, 40]. However, despite the increasing protective role of school-related parental support on trends in mental health problems over time, specifically PSS and DS, in both low and high SES groups, the problems have shown increasing trends in the low SES group while decreasing in the high SES group. While predicting the reasons behind this finding poses challenges, the vulnerability hypothesis could elucidate the observed situation, suggesting the presence of time-dependent shifts in the significance of risk or protective factors or the role of other factors in play. This becomes particularly noticeable in light of societal changes that could accentuate the importance of certain elements over others [28].

Unlike school-related parental support, teachers’ support did not have a significant association with either the trends or cross-sectional occurrence of adolescent mental health problems in this study. Previous studies have explained the precedence of parental support over teachers’ support regarding adolescents’ academic achievement, and also its influence on mental health, by the stronger bonds between parents and children, trust, communications, and engagement in adolescent academic life [44, 45]. Establishing effective partnership between parents and teachers, however, would synergize the effects of the support these parties provide to the adolescents [46].

The other remarkable finding was the increase of the protective role of participation over time on trends in SA among adolescents in the low SES group. This finding is consistent with previous research that attributed reduced risk of suicide among young people, among other things, to an increased sense of belongingness as a result of involvement in school-related issues [47]. This has an important practical implication, where schools can contribute to solving societal challenges through enhanced participation of students in decisions related to school matters, especially among low SES adolescents, who have a relatively lower civic engagement than those in the high SES group [48]. This means that, although participation was reported in previous research to have only a limited or no effect on well-being [49], it might be of particular importance in reducing the risk of SA among low SES groups.

Finally, one of the major findings in this study is that the protective association between school liking and all the mental health problems investigated in this study, except SI, became stronger over time. Different explanations for this relationship may emerge from interplays between different school-related factors. For example, previous studies found that students may dislike school due to difficulties with schoolwork or negative social relationship at school whereas, on the other hand, good social support helps overcome stress due to schoolwork as well as increasing school liking [7, 16, 17]. Given that the present study also found a increasing protective association between social support, such as school-related parental support, and mental health problems over time, it can be hypothesized that the protective association between school liking and adolescents´ mental health problems may similarly experience a growing effect.

In summary, higher scores of school-related in this study were found to be cross-sectionally associated with decreased mental health problems among adolescents in both SES groups. However, the increase or improvement in school-related factors is associated with decreased trends in mental health problems over time mainly among those in the high SES group. This highlights that the adolescents in the low SES group did not experience the same benefit from increased exposure to school-related factors over time as their counterparts in the high SES group did. In conclusion, this reinforces the role of SES in determining the long-term vulnerability disparities among these groups [25]. While the vulnerability hypothesis [21, 28] may explain this difference, further studies are necessary to delve into why trends in mental health problems differed along the SES gradients despite similar trends in school-related factors within both groups. More studies, similar to Högberg [20], for example, that go beyond school premises and consider changes at both macro and micro economic levels to investigate associations between school-related factors and mental health problems are needed to provide a comprehensive picture of the situation.

This study, however, has some limitations. As the result of a small number of participants in the low SES group, our data did not have enough power to test the significance of difference between the trends in mental health problems among the high and low SES groups. The descriptive presentation of differences in the trends, however, motivates further studies that can bridge this gap. Another limitation is that we could not find data for the whole study period (2004–2020) on all the indicators. We analyzed trends in PSS from 2004 to 2014, DS from 2004 to 2012, SI from 2004 to 2012, and SA from 2004 to 2020. This, however, offers an opportunity to present trends in more indicators over different time periods rather than focusing on only a few that have datapoints throughout an entire study period. Furthermore, this study relies on self-reported perceptions of school-related factors, which can potentially be influenced by participants’ mental health status. However, the study does not account for the impact of mental health status on students’ perceptions of these factors. This highlights the importance of future research to address this and other limitations in their investigations.

On the other hand, this study has several strengths. To our knowledge, it is the first to investigate the positive influences of various aspects of school context on mental health across SES gradients. It also spans a period of 16 years and uses data from a large sample of participants, which guarantees power for the testing of trends. Furthermore, this study has investigated trends in multiple aspects of mental health that might have been impacted differently by trends in different school-related factors.

## Conclusions

The results of this study showed robust protective cross-sectional associations between school-related factors and adolescent mental health problems. Nevertheless, we also found that the factors had varying degrees of influence depending on SES. Notably, in the low SES group, where increased trends in mental health problems were observed, only parental support and participation had remarkable protective association with trends in mental health problems over time. The absence of such protective associations between the rest of the factors and trends in mental health problems among adolescents in the low SES group may partially explain the increasing trends of the problems in this group. This calls for the need to promote and strengthen the school’s supportive role in young people’s development, with a focus on parental support and participation among adolescents with low SES to reduce inequities in young people’s mental health.

## Supporting information

S1 File (DOCX)

S2 File (DOCX)

S1 Fig Mean score of school factors by SES over years. a) School liking b) Participation c) Parental school support d) Teachers’ support, e) School physical environment.
(TIF)
