# Supplementary material for: Trends in mental health problems among Swedish adolescents: Do school-related factors play a role?
Source: PLoS One. 2024 Mar 8;19(3):e0300294. doi: 10.1371/journal.pone.0300294 (PMC10923405; doi:10.1371/journal.pone.0300294)
Supplement: S1 File — (DOCX) [file pone.0300294.s001.docx]

**Supporting information for manuscript:**

Trends in mental health problems among Swedish adolescents: do school-related factors play a role? Benti Geleta Buli, Peter Larm, Kent W. Nilsson, Charlotta Hellström-Olsson, Fabrizia Giannotta

*S1 Table. Total number of participants by survey year, sex, and SES*

| Year of survey | Data available during survey year^†^ | | | | Total | Girls (%) | High SES (%) | Response (%)^††^ |
| --- | --- | --- | --- | --- | --- | --- | --- | --- |
|  | PSS | DS | SI | SA |  |  |  |  |
| 2004 | Y | Y | Y | Y | 2899 | 49.5 | 79.6 | 80.0^a^ |
| 2006 | Y | Y | Y | Y | 3207 | 49.2 | 82.8 | 80.3^b^ |
| 2008 | Y | Y | Y | Y | 2690 | 49.4 | 85.4 | 78.2^c^ |
| 2010 | Y | Y | Y | Y | 2622 | 49.7 | 84.8 | 82.7^d^ |
| 2012 | Y | Y | Y | Y | 2121 | 49.2 | 86.4 | 82.0^e^ |
| 2014 | Y | N | N | N | 2211 | 50.9 | 87.6 | 87.0^f^ |
| 2017 | N | N | N | Y | 2006 | 50.8 | 86.4 | 75.0^g^ |
| 2020 | N | N | N | Y | 2117 | 52.3 | 89.0 | 77.8^h^ |
| Total |  |  |  |  | 19 873 | 50.0 | 84.8 | 80.4 |
| ^†^ PSS = psychosomatic symptoms; DS=depressive symptoms; SI: Suicide ideations; SA: Suicide attempts; Y=Yes, N=No  ^††^ **Sources of response rates information**: (a) Åslund, et al., 2007; (b) Åslund, et al., 2009; (c) Hellström, et al., 2015; (d) Västmanland Regional report, 2010; (e) Kerstis, et al., 2012; (f) Västmanland Regional report, 2014; (g) Institute for Quality Indicators in Västmanland, 2017; (h) Västmanland Regional report, 2020 | | | | | | | | |

*S2 Table. Mean scores of outcome variables, their standard deviations, and proportion of missing values, with changes in mean scores over time tested using Reverse Helmert Contrast§*

| **Survey year** | **PSS (N=15,750)** | | **DS (N=13,539)** | | **SI (N=12,972)** | | **SA (N=16,073)** | |
| --- | --- | --- | --- | --- | --- | --- | --- | --- |
|  | **Low SES**  ***Mean (S.D)*** | **High SES**  ***Mean (S.D)*** | **Low SES**  ***Mean (S.D)*** | **High SES**  ***Mean (S.D)*** | **Low SES**  ***Mean (S.D)*** | **High SES**  ***Mean (S.D)*** | **Low SES**  ***Mean (S.D)*** | **High SES**  ***Mean (S.D)*** |
| 2004 | 12.0 (6.19) | 11.5 (5.78) | 3.3 (2.73) | 2.9 (2.61) | 0.19 (0.39) | 0.16 (0.37) | 0.15 (0.36) | 0.11 (0.31) |
| 2006 | 14.0 (6.43)* | 11.0 (5.61)^#^ | 4.1 (2.98)* | 2.7 (2.69)^#^ | 0.25 (0.44)* | 0.16 (0.36) | 0.27 (0.44)* | 0.13 (0.34)* |
| 2008 | 14.4 (6.42)* | 11.8 (5.67)* | 4.3 (2.93)* | 2.8 (2.68) | 0.24 (0.43) | 0.13 (0.33)^#^ | 0.22 (0.42) | 0.09 (0.28)^#^ |
| 2010 | 13.6 (6.23) | 10.9 (5.68)^#^ | 4.3 (2.98) | 2.8 (2.69) | 0.24 (0.43) | 0.13 (0.34) | 0.22 (0.41) | 0.09 (0.29)^#^ |
| 2012 | 13.9 (6.80) | 11.2 (5.53) | 4.2 (2.96) | 2.5 (2.61)^#^ | 0.20 (0.40) | 0.12 (0.33)^#^ | 0.24 (0.43) | 0.07 (0.26)^#^ |
| 2014 | 12.4 (6.49)^#^ | 10.3 (5.78)^#^ | DNA | DNA | DNA | DNA | DNA | DNA |
| 2017 | DNA | DNA | DNA | DNA | DNA | DNA | 0.21 (0.41) | 0.09 (028) |
| 2020 | DNA | DNA | DNA | DNA | DNA | DNA | 0.21 (0.41) | 0.11 (031) |
| Overall | 13.3 (6.45) | 11.1 (5.69) | 4.0 (2.93) | 2.8 (2.66) | 0.23 (0.42) | 0.14 (0.35) | 0.21 (0.41) | 0.10 (0.30) |
| Missing (%) | 1.4 | | 0.0 | | 4.2 | | 9.0 | |
| PSS =psychosomatic symptoms, DS=depressive symptoms, SI=suicidal ideations, SA=Suicidal attempts, DNA=Data not available. * = significant increase (at p<0.05) in mean score compared to average of previous means; # = significant decrease (at p<0.05) in mean score compared to average of previous mean scores. §: Reverse Helmert Contrast is a statistical technique that compares the value of a variable at given level with the mean of the previous level(s). *(Reference: Schad DJ, Vasishth S, Hohenstein S, Kliegl R. How to capitalize on a priori contrasts in linear (mixed) models: A tutorial. Journal of Memory and Language. 2020; 110:104038).* | | | | | | | | |

S3 Table. Pattern Factor Loadings for Exploratory Factor Analysis of school-related factors (N = 13,985).

| **Item No.** | **Items** | **Factors** | | |
| --- | --- | --- | --- | --- |
|  |  | **1** | **2** | **3** |
| 1 | If I have problems at school my parents are ready to help me | 0.84 |  |  |
| 2 | My parents encourage me to do well in school | 0.79 |  |  |
| 3 | If the schoolwork is difficult, I can get help from my parents | 0.77 |  |  |
| 4 | The teachers give me useful viewpoints on my work |  | 0.86 |  |
| 5 | The teachers are good at making us think for ourselves |  | 0.77 |  |
| 6 | The teachers make efforts to ensure that no student is discriminated or bullied |  | 0.70 |  |
| 7 | There is plenty of space in the classrooms |  |  | 0.83 |
| 8 | The classrooms are clean and pleasant |  |  | 0.72 |
| 9 | The classrooms are calm and enhance peace of mind |  |  | 0.67 |
|  | *Eigenvalues* | 3.73 | 1.72 | 1.16 |
|  | *% Variance explained (73.6%)* | 41.63 | 19.12 | 12.86 |
|  | Kaiser-Meyer-Olkin (KMO) test for sample adequacy | 0.82 | | |
|  | Bartlett's test of sphericity | P=0.00 | | |
|  | Factor correlations:   1. Factor 1 Vs Factor 2 2. Factor 1 Vs Factor 3 3. Factor 2 Vs Factor 3 | 0.403  0.295  0.538 | | |
|  | Extraction Method: | Principal Axis Factoring | | |
|  | Rotation type: | Promax | | |
