# Supplementary material for: Trends in mental health problems among Swedish adolescents: Do school-related factors play a role?
Source: PLoS One. 2024 Mar 8;19(3):e0300294. doi: 10.1371/journal.pone.0300294 (PMC10923405; doi:10.1371/journal.pone.0300294)
Supplement: S2 File — (DOCX) [file pone.0300294.s002.docx]

**Supporting information for manuscript:**

Trends in mental health problems among Swedish adolescents: do school-related factors play a role?

Benti Geleta Buli, Peter Larm, Kent W. Nilsson, Charlotta Hellström-Olsson, Fabrizia Giannotta

S4 Table. Mean scores and standard deviations of school-related factors disaggregated by socio economic status (SES).

| **Variables** | **SES** | **2004 - M(SD)‡** | **2006 - M(SD)** | **2008 - M(SD)** | **2010 - M(SD)** | **2012 - M(SD)** | **2014 - M(SD)** | **2017 - M(SD)** | **2020 - M(SD)** |  |
| --- | --- | --- | --- | --- | --- | --- | --- | --- | --- | --- |
| Parental support | Low | 12.02 (3.06) | 11.69 (3.53) | 11.58 (3.39) | 11.56 (3.31) | 11.89 (3.27) | 11.50 (3.08) |  |  |  |
|  | High | 12.98 (2.72) | 11.67 (3.4^)^ | 13.13 (2.53) | 13.05 (2.71) | 13.31 (2.42) | 13.09 (2.40) |  |  |  |
| Teachers’ support | Low | 10.95 (2.98) | 10.14 (3.44) | 10.57 (3.07) | 10.18 (3.30) | 10.90 (3.18) | 10.69 (2.87) |  |  |  |
|  | High | 11.04 (3.03) | 10.14 (3.29) | 10.89 (2.99) | 11.02 (3.07) | 11.74 (2.81) | 11.17 (2.75) |  |  |  |
| School physical environment | Low | 9.49 (2.82) | 8.70 (3.10) | 8.93 (2.91) | 9.24 (2.90) | 9.98 (2.81) | 9.84 (2.86) |  |  |  |
|  | High | 9.71 (2.89) | 9.24 (3.03) | 9.52 (2.81) | 9.96 (2.69) | 10.29 (2.77) | 10.48 (2.62) |  |  |  |
| School liking | Low | 3.70 (1.08) | 3.77 (1.07) | 3.50 (1.13) | 3.67 (1.07) | 3.69 (1.02) | 3.73 (1.06) | 3.40 (1.08) | 3.29 (1.09) |  |
|  | High | 3.94 (0.96) | 3.86 (1.02) | 3.93 (0.96) | 4.01 (0.91) | 4.07 (0.90) | 4.13 (0.89) | 3.90 (0.91) | 3.78 (1.00) |  |
| Participation in decision making | Low | 2.58 (1.12) | 2.59 (1.16) | 2.55 (1.07) | 2.63 (1.14) | 2.73 (1.10) |  |  | 3.22 (1.08) |  |
|  | High | 2.61 (1.11) | 2.70 (1.12) | 2.71 (1.11) | 2.78 (1.11) | 2.78 (1.12) |  |  | 3.37 (1.10) |  |
| ‡ M = Mean score; SD = standard deviation. | | | | | | | | | | |

S5 Table. Mean scores of socioeconomic statuses (SES) reported by students by year of survey, compared using Reverse Helmert Contrasts

| Year of survey | Mean (95% CI) | Difference (mean of current year vs mean of previous means) |
| --- | --- | --- |
| 2004 | 3.53 (3.49, 3.57) | - |
| 2006 | 3.43 (3.39, 3.47) | -0.10*** |
| 2008 | 3.39 (3.35, 3.44) | -0.09** |
| 2010 | 3.42 (3.38, 3.46) | -0.03 |
| 2012 | 3.46 (3.41, 3.51) | 0.02 |
| 2014 | 3.66 (3.61, 3.71) | 0.21*** |
| 2017 | 3.74 (3.68, 3.79) | 0.25*** |
| 2020 | 3.70 (3.65, 3.75) | 0.18*** |
| 2004 vs 2020 |  | 0.17***  *(Cohen’s d = 0.147)* |
| ***/** = p<.001/.01 respectively. CI = Confidence Interval  F = 31.982, df=7, P<0.001, eta^2^ = .012 | | |

S6 Table. Direct and indirect effects of year of survey on mean scores/probabilities of mental health problems, through school-related factors (school liking, participation, parental support, teacher’s support, and school physical environment). Results are presented in unstandardized coefficients.

|  | Low SES | | High SES | |
| --- | --- | --- | --- | --- |
|  | Effect (SE) | LL – UL | Effect (SE) | LL – UL |
| **a)      Psychosomatic symptoms** |  |  |  |  |
| Year of survey (direct) – (d) | 0.401 (0.098) | 0.210 – 0.593 | 0.036 (0.040) | -0.042 – 0.113 |
| School liking | 0.015 (0.018) | -0.019 – 0.053 | **-0.038 (0.007)** | **-0.051 – -0.025** |
| Participation | 0.008 (0.007) | -0.002 – 0.024 | 0.005 (0.002) | 0.000 – 0.010 |
| Parental support | 0.022 (0.016) | -0.007 – 0.055 | **-0.023 (0.005)** | **-0.034 – -0.014** |
| Teacher’s support | 0.009 (0.008) | -0.003 – 0.028 | **-0.011 (0.005)** | **-0.021 – -0.003** |
| School physical environment | -0.024 (0.014) | -0.054 – 0.001 | **-0.036 (0.006)** | **-0.048 – -0.025** |
| Total indirect effect – (i) | 0.030 (0.038) | -0.042 – 0.106 | -0.104 (0.012) | -0.126 – -0.081 |
| Total effect (t = d + i) | **0.432 (0.104)** | **0.228 – 0.635** | -0.068 (0.041) | -0.148 – 0.012 |
| **b)      Depressive symptoms** |  |  |  |  |
| Year of survey (direct) – (d) | 0.233 (0.044) | 0.146 - 0.319 | -021 (0.019) | -0.057 – 0.016 |
| School liking | 0.007 (0.008) | -0.009 – 0.024 | **-.022 (0.004)** | **-0.029 – -0.015** |
| Participation | 0.003 (0.003) | -0.000 – 0.010 | -0.001 (0.001) | -0.002 – 0.002 |
| Parental support | 0.014 (0.009) | -0.004 – 0.032 | **-0.021 (0.003)** | **-0.027 – -0.016** |
| Teacher’s support | 0.003 (0.003) | -0.001 – 0.010 | -0.004 (0.002) | -0.008 – 0.000 |
| School physical environment | -0.009 (0.005) | -0.020 – 0.001 | **-0.009 (0.002)** | **-0.014 – -0.005** |
| Total indirect effect – (i) | 0.019(0.018) | -0.015 – 0.053 | **-0.056 (0.006)** | **-0.067 – -0.045** |
| Total effect (t = d + i) | **0.252 (0.047)** | **0.159 – 0.344** | -0.076 (0.019) | -0.114 – 0.001 |
| **c)       Suicidal ideations** |  |  |  |  |
| Year of survey (direct) – (d) | 0.008 (0.042) | -0.074 - 0.090 | **-0.065 (0.022)** | **-0.108 – -0.021** |
| School liking | 0.004 (0.005) | -0.005 – 0.014 | **-0.013 (0.002)** | **-0.018 – -0.008** |
| Participation | 0.002 (0.002) | -0.002 – 0.007 | -0.001 (0.001) | -0.004 – 0.001 |
| Parental support | 0.009(0.006) | -0.003 – 0.022 | **-0.015 (0.002)** | **-0.020 – -0.011** |
| Teacher’s support | 0.004 (0.003) | -0.001 – 0.011 | -0.002 (0.002) | -0.007 – 0.003 |
| School physical environment | -0.003 (0.003) | -0.011 – 0.001 | -0.002 (0.002) | -0.007 – 0.002 |
| Total indirect effect – (i) | 0.015 (0.012) | -0.015 – 0.053 | **-0.033 (0.004)** | **-0.041 – -0.026** |
| Total effect (t = d + i) ^±^ | 0.023 |  | -0.098 |  |
| **d)      Suide attaempts** |  |  |  |  |
| Year of survey (direct) | 0.022 (0.028) | -0.033 – 0.076 | -0.034 (0.015) | -0.064 – 0.004 |
| School liking | 0.021 (0.006) | 0.010 – 0.033 | **0.004 (0.002)** | **0.0002 – 0.008** |
| Participation | -0.002 (0.004) | -0.010 – 0.007 | -0.002 (0.003) | -0.009 – 0.004 |
| Total indirect effect | 0.019 (0.008) | 0.005 – 0.034 | 0.002 (0.004) | -0.006 – 0.009 |
| Total effect (t = d + i) ^±^ | 0.041 |  | -0.032 |  |
| ^±^In Hayes’ process, total effect model is not available with dichotomous outcome. | | | | |

S7 Table. Logistic regression results of the association between school-related factors and SA (2004-2012) among adolescents aged about 15 years.

| Variables | **Low SES (i) (n=2,178)**  OR (95% CI) | | **High SES (j) (n=11,072)**  OR (95% CI) | |
| --- | --- | --- | --- | --- |
|  | Model 1 | Model 2 | Model 1 | Model 2 |
| School liking | **.739** (.658, .829) | **.730** (.649, .820) | **.732** (.680, .789) | **.716** (.664, .772) |
| Participation | 1.07 (.962, 1.20) | 1.07 (.955, 1.20) | .988 (.925, 1.06) | .978 (.914, 1.05) |
| Parent support in school | **.898** (.866, .932) | **.897** (.864, .930) | **.876** (.856, .897) | **.866** (.846, .887) |
| Teachers' support | .965 (.924, 1.02) | .967 (.926, 1.01) | .998 (.972, 1.03) | 1.00 (.973, 1.03) |
| School environment | .966 (.922, 1.01) | .961 (.916, 1.01) | .991 (963, 1.02) | .998 (.969, 1.03) |
| Year of survey | 1.09 (1.00, 1.19) | 1.04 (.946, 1.15) | **.907** (.860, .957) | **.865** (818, .914) |
| Sex (girl) | **1.48** (1.16, 1.89) | **1.48** (1.16, 1.89) | **1.80** (1.55, 2.08) | **1.81** (1.57, 2.10) |
| School liking * year |  | .991 (.910, 1.80) |  | **.919** (.867, .974) |
| Participation* year |  | .971 (894, 1.05) |  | .972 (.924, 1.02) |
| Parent support * year |  | .987 (.960, 1.01) |  | **.948** (.931, .965) |
| Teacher support * year |  | .996 (.964, 1.03) |  | 1.00 (.983, 1.03) |
| School env. * year |  | .965 (.931, 1.00) |  | 1.02 (.998, 1.04) |
| OR = Odds ratio (*significant values at α<.05 are presented in bold*), CI = 95% Confidence Interval | | | | |
