# Supplementary figures and images for: Trends in mental health problems among Swedish adolescents: Do school-related factors play a role?
Source: PLoS One. 2024 Mar 8;19(3):e0300294. doi: 10.1371/journal.pone.0300294 (PMC10923405; doi:10.1371/journal.pone.0300294)

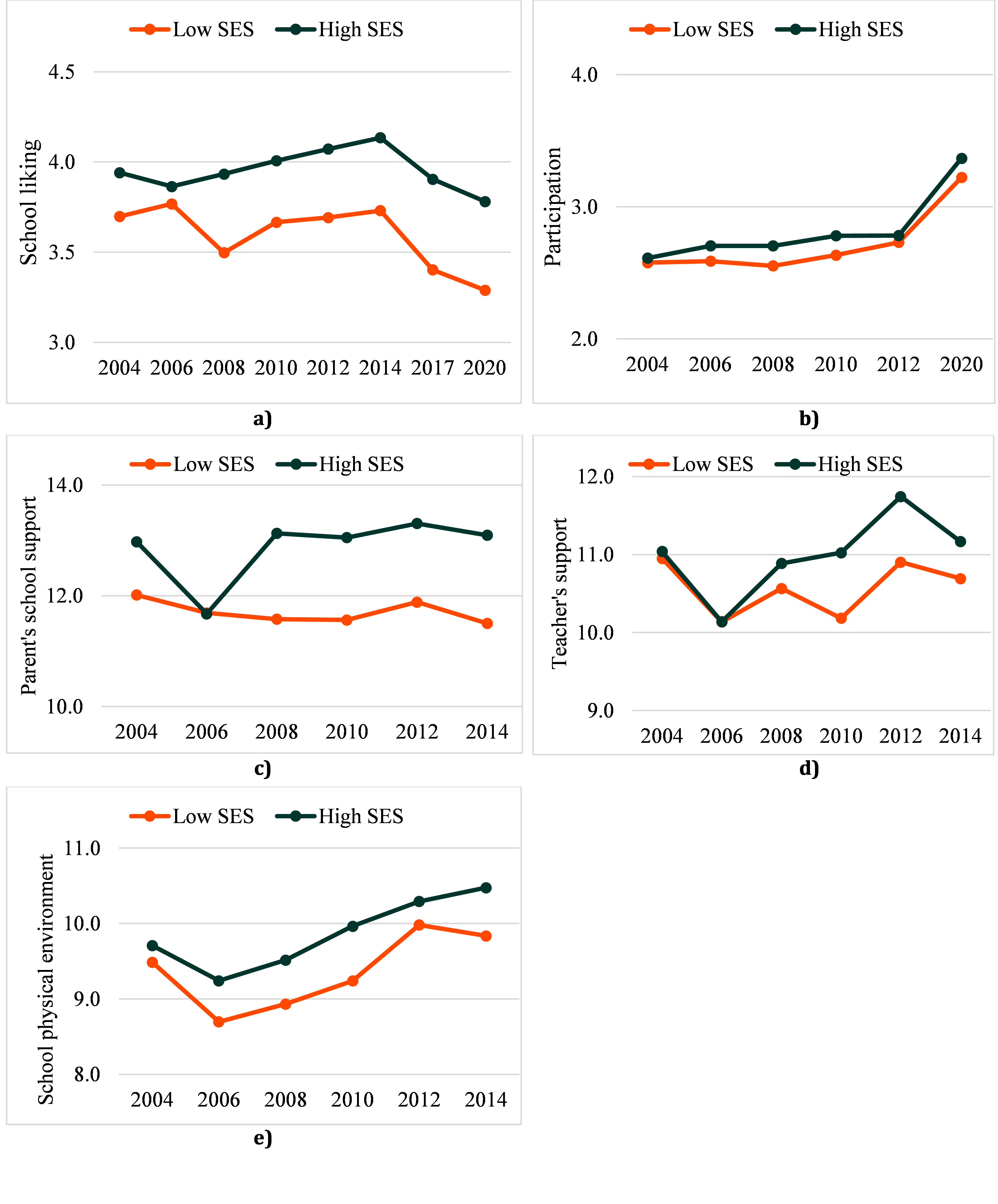

Supplement: S1 Fig — a) School liking b) Participation c) Parental school support d) Teachers’ support, e) School physical environment. (TIF) [file pone.0300294.s003.tif]
